# Targeted multi-epitope therapeutic vaccine for the treatment of invasive glioblastoma multiforme

**DOI:** 10.1186/2051-1426-3-S2-P436

**Published:** 2015-11-04

**Authors:** Xiaofang Huang, Rashida Ginwala, Aykan Karabudak, Pooja Jain, Ramila Philip

**Affiliations:** 1 Immunotope Inc., Doylestown, PA, USA; 2 Drexel University College of Medicine, Philadelphia, PA, USA

## 

Invasive glioblastoma multiforme (GBM) is the most common malignant primary tumor of the central nervous system with an estimated annual incidence of 3.15 cases per 100,000 in the USA. Current therapies have been unsuccessful in curing malignant gliomas that are highly proliferative and infiltrating, containing regions of necrosis and hypervascularization. Novel therapeutic approaches are critically needed. A glioma specific immune response offers a potential anti-tumor benefit by immune targeted elimination of tumor cells and memory capability that may prevent future recurrence, a feature of aggressive GBM. A number of GBM tumor associated antigens (TAAs) have been studied in preclinical and clinical studies. However, most of these TAAs are HLA motif predicted T cell epitopes derived from over-expressed proteins. A systematic analysis of the naturally processed and therefore most clinically relevant MHC class I associated T cell epitopes presented by glioma cells has never been reported. A number of tumor associated over expressed glycoproteins have been identified as biomarkers and have been proposed as a potential immunotherapeutic target for various cancers including GBM. Tumorigenesis involves dysregulation such as over production or high turnover of proteins that are involved in tumor initiation, progression, unlimited growth, invasion, immune evasion, therapy resistance and survival. One of the key features of dysregulation is aberrant glycosylation of glycoproteins that are involved in these cancer pathways. In this study, we attempt to identify and characterize T cell epitopes presented on the U87 human glioblastoma cells and select epitopes that are derived from glycosylated proteins to develop a multi-epitope based therapeutic vaccine product that induce specific T cell responses against lethal GBM. Towards this goal, we enriched GlcNAc expressing glycoproteins using L-PHA lectin and identified total of 66 glycopeptides derived from 59 different glycoproteins with aberrant GlcNAc glycosylation from U87 cells via glycoproteomic analysis. We also used an immunoproteomic approach to investigate MHC class I presented peptides in tumor cells. Those selected MHC class I epitiopes were derived from glycosylated proteins that had been identified previously. In addition, these peptides were able to activate CD8+ T cell responses against glioma cells. Thus the selection of T cell epitopes derived from aberrantly glycosylated proteins forms the foundation for the development of a multi-epitope based therapeutic vaccine that induces specific T cell responses against lethal GBM.

